# Primary stability of a shoulderless Zweymüller hip stem: a comparative in vitro micromotion study

**DOI:** 10.1186/s13018-016-0410-1

**Published:** 2016-07-05

**Authors:** Ralf Bieger, Tobias Freitag, Anita Ignatius, Heiko Reichel, Lutz Dürselen

**Affiliations:** Department of Orthopaedic Surgery, Centre of Musculoskeletal Research, University Hospital Ulm, Oberer Eselsberg 45, 89081 Ulm, Germany; Institute of Orthopaedic Research and Biomechanics, Centre of Musculoskeletal Research, University of Ulm, Ulm, Germany

**Keywords:** Total hip arthroplasty, Primary stability, Cadaver, Stress, Mechanical, Micromotion

## Abstract

**Background:**

The Zweymüller stem design has proven long-term stability with a 20-year survival rate of over 90 %. Primary stability necessitates implant-bone micromotions below 150 μm, otherwise bony ingrowth is negatively influenced.

**Methods:**

Using fresh paired human femurs, we investigated a modification of the Zweymüller-type stem design with reduced proximal lateral shoulder in reference to primary stability. Relative motion between the implant and the cortical bone as well as the irreversible implant migration was investigated under dynamic loading (100–1600 N) over 100,000 cycles using miniature displacement transducers.

**Results:**

Micromotions were below the critical threshold for both implants at all measurement points. Axial reversible and irreversible micromotions were not influenced by reducing the shoulder of the prosthesis. Resistance against rotational moments was less pronounced after reduction of the shoulder without statistical significant results.

**Conclusions:**

Reducing the proximal shoulder of the Zweymüller-type stem design does not negatively influence axial stability but might negatively influence rotational stability. Even though, comparable results still suggest a reasonable resistance against rotational forces.

## Background

Cementless as well as cemented total hip arthroplasty (THA) demonstrated long-term survival rates after 15 years of over 90 % [1]. Nowadays, the majority of THA surgeries are performed without cement [2]. Aseptic loosening, due to the absence of primary stability, wear, and periprosthetic bone loss as a result of the implant-specific bone remodelling are the main reasons for implant failure [1]. Primary stability comprises reversible implant-bone micromotion as well as irreversible implant migration [3]. However, relative implant-bone micromotions larger than 150 μm can compromise bony ingrowth of porous coated implants leading to fibrous tissue in the interface, investigated by animal studies [4, 5]. Initial stability of cementless implants is achieved by press-fitting of the prosthesis in the femur [2]. Khanuja et al. established a classification in reference to the region and concept of the mode of fixation [2]. They classified a total of six different design concepts with evidence of excellent long-term results. However, most of these stems do not allow soft tissue and bone-sparing surgery which is more and more important in young and active patients with end-stage hip disease [6].

According to the Zweymüller philosophy, the stem is tapered, cementless, with a rectangular cross section and a four-point fixation providing rotational stability in the metaphyseal and diaphyseal region [2]. Recently, 20-year survival of the Alloclassic Zweymüller stem (Zimmer, Winterthur, Switzerland) of 96 % was described [6]. Nevertheless, the authors stated one disadvantage of the prosthesis, the extensive shoulder of the implant, which should provide rotational stability but does not allow tissue-sparing surgery [6]. Another problem with shouldered implants and most straight stems is a thinning of the proximal lateral femur which can result in avulsion fractures of the major trochanter causing abductor weakness and gluteal pain [7]. Therefore, these implants should be adapted for the needs of less invasive surgery. However, changes of the design of an implant can affect the biomechanical performance of a prosthesis including primary stability [8].

In this in vitro study, we investigated a modification of the Zweymüller stem design with reduced proximal lateral shoulder with regard to primary stability. The hypothesis of this investigation was that reducing the proximal lateral shoulder of the Zweymüller straight stem would not negatively influence primary stability due to the press fit concept in the proximal diaphyseal region.

## Methods

**Fig. 1:**
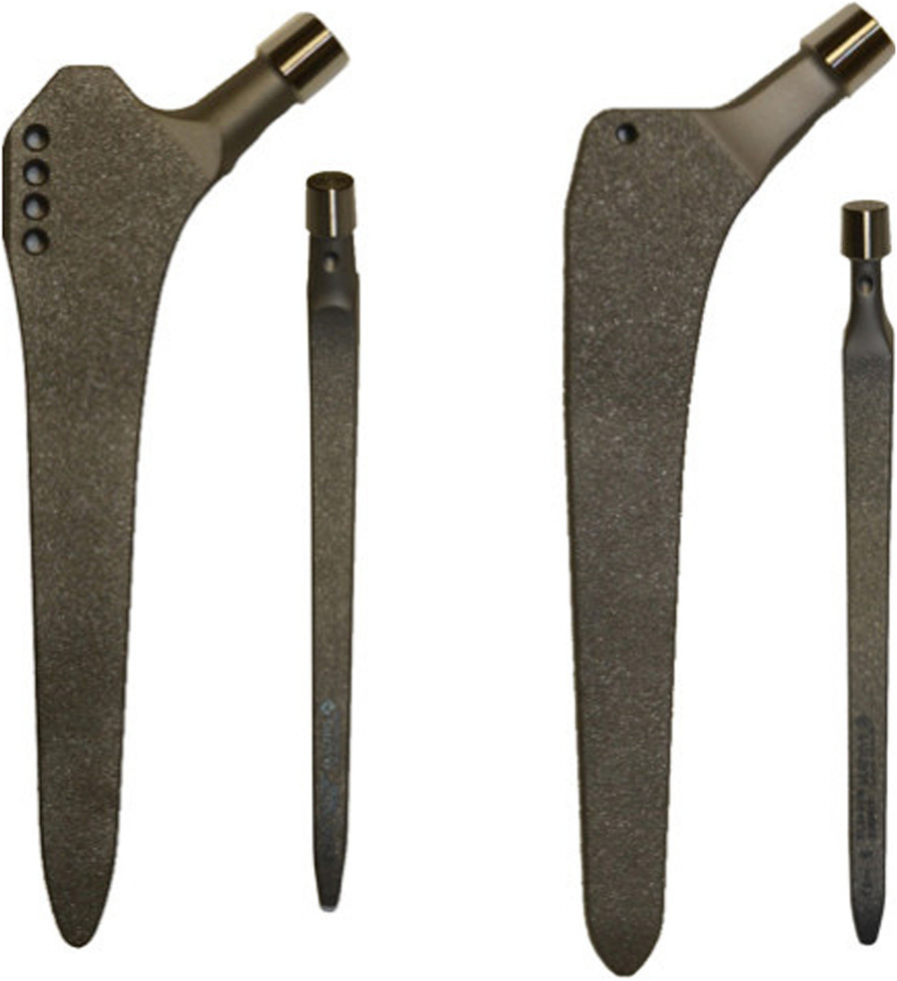
Anterior and medial profile of the CBH (*left*) and the CBH bone preserving stem (*right*)

The two prostheses used in this study were the two Zweymüller-type straight stems, CBH and the CBH bone preserving (both Mathys medical, Bettlach, Switzerland; Fig. 1). Both stems are made of a titanium alloy designed for cementless implantation. The surfaces are rough blasted to promote osseous integration. Each stem is available with two different offset options to allow restoration of the individual hip geometry.

The CBH is a Zweymüller-type straight stem with a rectangular cross section and a tapered design. Press-fitting and rotational stability is predominantly attained in the proximal diaphyseal region. The original idea included rotational stability supported by a lateral proximal wing-shaped shoulder. The CBH bone preserving (bp) is designed for less invasive surgery respecting the bone stock in the proximal femur. For this reason, the proximal lateral shoulder of the prosthesis was reduced and the tip of the prosthesis was flattened (Fig. 1).

After approval from the Institutional Review Board of the University of Ulm, six pairs of fresh-frozen human femurs were obtained via ScienceCare (Phoenix, AZ, USA). Three donors were female and three male, with a mean age of 38 years (range 19–52 years). Malignant tumours, bone-quality-affecting diseases, or fractures were ruled out with radiographs in two planes. The estimated size of the prosthesis was templated on digital, scaled radiographs performed before implantation of either the CBH or CBH bp.

**Fig. 2:**
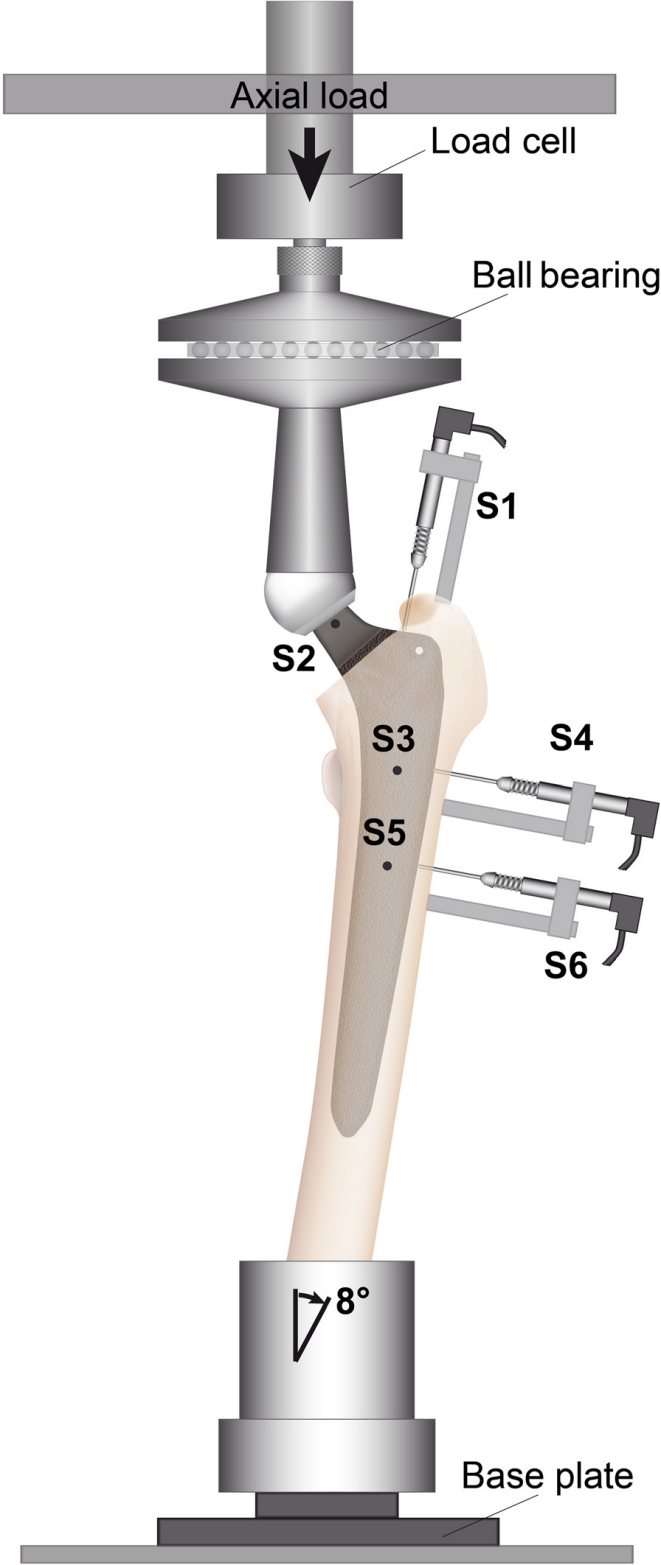
Illustration of the test set-up. S1–S6 demonstrate the locations of the six inductive miniature displacement transducers

The experimental design has been described previously [9]. In brief, the basic preparation of the specimens included soft tissue removal and shortening to an equal length of 37 cm below the greater trochanter. Neck anteversion was recorded before cutting the femoral condyles for subsequent orientation. Furthermore, the resected femurs were fixed in a steel cup using methylmethacrylate (Technovit 3040; Heraeus Kulzer, Wehrheim, Germany). One of each type of prosthesis was implanted by an experienced orthopaedic surgeon in either the right or the left of each pair of femurs. For measurement of the relative motion between the implant and the cortical bone, we attached a total of six inductive miniature displacement transducers (HBM WI/5mm-T; HBM, Darmstadt, Germany) with a precision of 1 μm to the bone. Relative axial implant-bone motion was measured at transducer S1 at the shoulder of the prosthesis (Fig. 2). Rotational stem motion was captured at transducer S2, which was attached perpendicular to the neck of the implant (Fig. 2). The measured micromotions were converted into rotation around the femoral axis by gauging the distance between the tip of the transducer and the longitudinal axis of the diaphysis of the femur. To ensure correct position of the implants along the femoral axis, X-rays in two planes were performed after implantation. Transducers S3–S6 measured implant-bone micromotion perpendicular to the implant. For this reason, 4-mm drill holes provided access between the tip of the device and the prosthesis. Two transducers were located at the level of the minor trochanter on the ventral (S3) and lateral (S4) side (Fig. 2). The transducers S5 (ventral) and S6 (lateral) were located 4 cm below the minor trochanter (Fig. 2). The femur was mounted in a servo hydraulic material testing machine (Instron, Type 8871, Pfungstadt, Germany), which applied a vertical load. A ball bearing was attached between the device and the load cell to achieve a moment-free introduction of the load (Fig. 2). A single-leg stance, creating bending as well as torsional moments, was simulated by tilting the femur 8° in the lateral direction in the frontal plane and by 6° dorsally in the sagittal plane [10]. The material testing machine applied 100,000 dynamic sinusoidal load cycles at a frequency of 2 Hz, which simulates the first six postoperative weeks [11]. Each cycle applied between 100 and 1600 N, which corresponds to approximately 2.5 times the body weight occurring during normal gait [10, 12]. Reversible implant-bone motion was captured every 500 cycles at all six devices. We analysed reversible micromotions after 40,000 and 100,000 loading cycles. Earlier studies showed no further changes in implant-bone micromotions after 40,000 loading cycles [3, 9]. Therefore, the amplitude of six consecutive cycles was averaged for each transducer. Furthermore, irreversible implant migration in the axial direction (S1) was calculated by the difference between the maximum deformation occurring during the first and the last cycle of the 100,000 loading cycles. In the same way, the irreversible torsion around the femoral axis was calculated at transducer S2.

Statistical analysis was performed using JMP statistical analysis software (SAS Institute, Gary, USA). The Wilcoxon signed-rank test was used to analyse differences between the two stems used. Significance was assumed for *p* ≤ 0.05.

## Results

Four devices failed before 100,000 loading cycles were applied. In the CBH group, one calcar crack occurred after about 50,000 cycles, and in one case, the ball bearing jumped out after about 40,000 loading cycles and no further implant-bone micromotion could be recorded. For the same reason, two femurs had to be excluded after 40,000 loading cycles in the CBH bp group. Therefore, reversible implant-bone micromotions could be recorded after 40,000 loading cycles in six specimens in each group and in four specimens in each group after 100,000 loading cycles.

After 40,000 loading cycles, the mean micromotion amplitudes were below 150 μm at all six transducer locations for both prostheses (Table 1). Mean implant-bone micromotions were between 6.6 μm (±3.3 μm; transducer S5) and 45.6 μm (±15.6 μm; transducer S2) in the CBH group and between 9.9 μm (±8.3 μm; transducer S5) and 67.1 μm (±45.7 μm, transducer S2) in the CBH bp group.

**Table 1:** Mean reversible implant-bone micromotion amplitudes (μm) and standard deviations (SD) after 40,000 loading cycles at transducers S1–S6 (both *n* = 6)

Transducer		CBH		CBH bp		*p* value
		Mean	SD	Mean	SD	
S1	μm	23.9	34.4	15.1	8.1	0.125
S2	μm	45.6	15.6	67.1	45.7	0.313
S3	μm	12.1	7.1	18.0	13.2	1.000
S4	μm	12.3	14.0	39.9	69.5	0.584
S5	μm	6.6	3.3	9.9	8.3	0.813
S6	μm	9.1	4.7	12.8	16.8	1.000

**Fig. 3:**
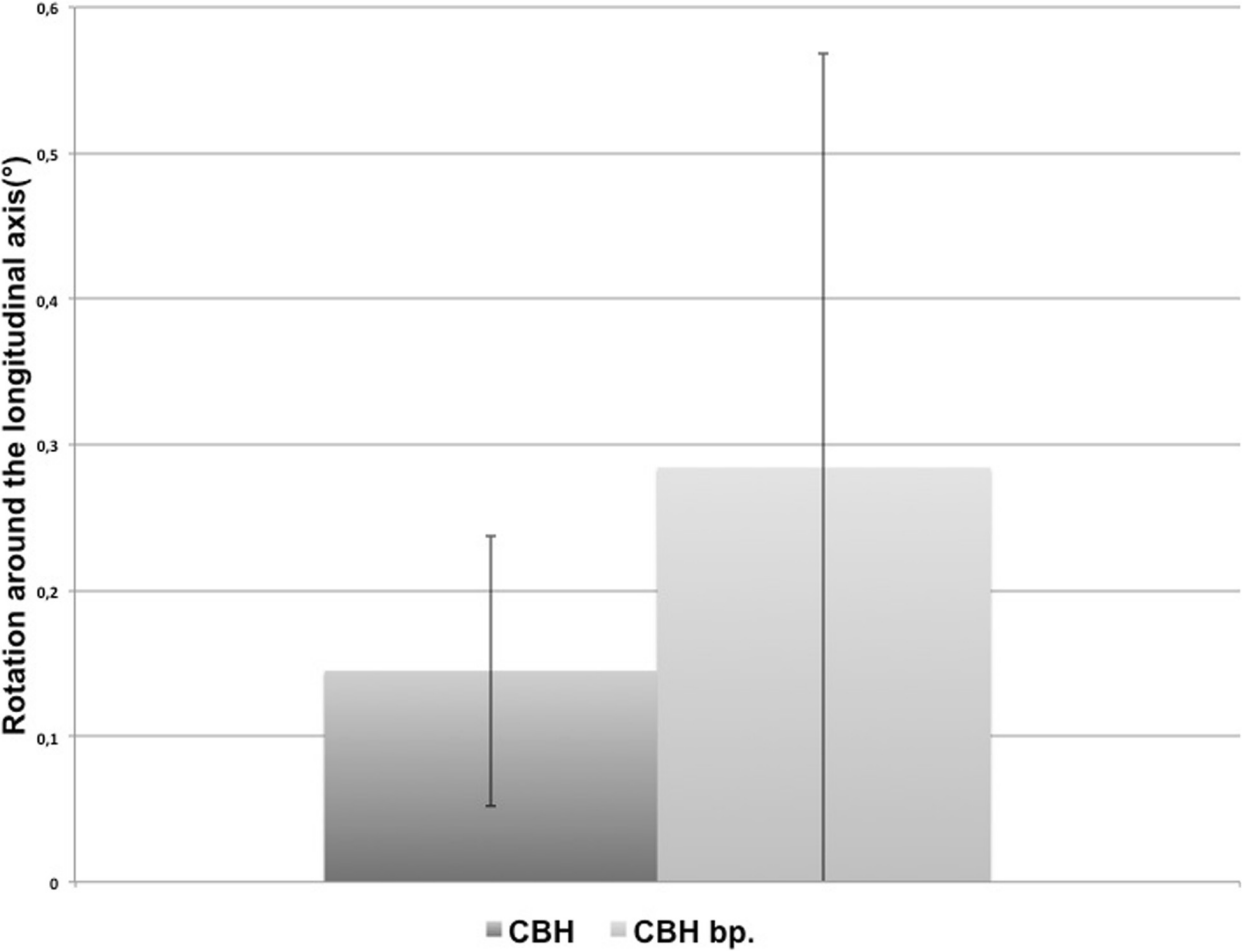
Illustration of the means of the calculated reversible rotation after 40,000 loading cycles around the longitudinal axis (°) for both stems (both *n* = 6)

The calculated rotation after 40,000 cycles around the femoral axis was in the retrotorsional direction for both implants. Reversible retrotorsion was 0.14° (±0.09°) in the CBH group and 0.28° (±0.28°) in the CBH bp group (Fig. 3).

**Fig. 4:**
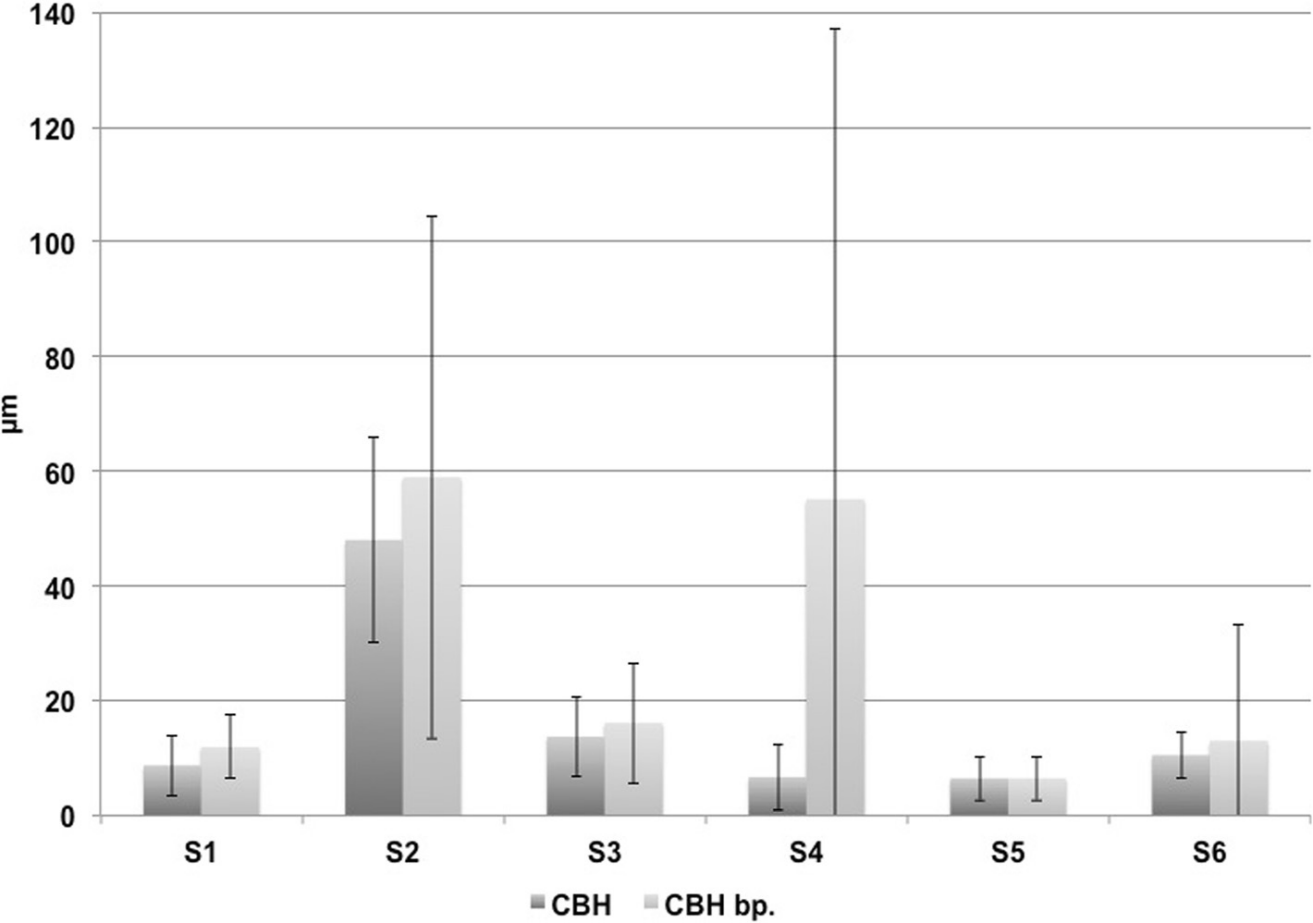
Illustration of the mean reversible implant-bone micromotion amplitudes (μm) for the CBH and CBH bp stems after 100,000 loading cycles (both *n* = 4) at transducers S1–S6

After 100,000 loading cycles, the mean micromotion amplitudes were between 6.4 μm (±3.9 μm; transducer S5) and 47.9 μm (±17.9 μm; transducer S2) in the CBH group and between 6.4 μm (±3.8 μm, transducer S5) and 58.9 μm (±45.6 μm; transducer S2) in the CBH bp group without statistical differences (Fig. 4). Mean retrotorsion was 0.12° (±0.11°) in the CBH group and 0.19° (±0.27°) in the CBH bp group (*p* = 0.88).

Axial irreversible migration after 40,000 loading cycles was 74 μm (±80 μm; *n* = 6) in the CBH group and 9.3 μm (±10.6 μm; *n* = 6) in the CBH bp group (*p* = 0.13) and after 100,000 loading cycles 37.7 μm (±36.6 μm; *n* = 4) in the CBH group and 2.1 μm (±14.6 μm; *n* = 4) in the CBH bp group (*p* = 0.25). Irreversible rotation towards retrotorsion was 0.04° (±0.69°; CBH; *n* = 6) compared to 0.59° (±0.41°; CBH bp; *n* = 6) after 40,000 loading cycles (*p* = 0.63) and 0.15° (±0.37°; CBH; *n* = 4) compared to 0.43° (±0.62°; CBH bp; *n* = 4) after 100,000 loading cycles.

## Discussion

Primary stability including reversible implant-bone micromotions as well as irreversible migration of a straight stem with reduced proximal shoulder and flattened tip was compared to a stem following the original design of the Zweymüller philosophy. In this in vitro study, both implants proved primary stability with micromotions well below the critical threshold of 150 μm [4, 5].

The concept of cementless fixation can be addressed with different design concepts [2]. The Zweymüller-type prosthesis is a clinically proven straight stem locking in the distal metaphysis and proximal diaphysis with a four-point fixation concept [2, 13]. Furthermore, the original developer described two main characteristics to resist torsional moments, the proximal leaflike shoulder and the cross-sectional shape of the stem with a four-point fixation in the femoral metaphysis and diaphysis [14]. Therefore, modifications of the shoulder of the prosthesis might influence the bone-implant stability. This context was demonstrated in a finite-element study describing the influence of even small changes of the geometry of an implant on the biomechanical behaviour [8]. In the current study, only small and non-significant changes on implant-bone micromotions at all six measurement points as well as irreversible migration especially in the axial direction were found. These findings might be explained by the distal locking mechanism of the stem which was shown in vivo with proximal bone atrophy as a result of stress shielding around stable Zweymüller stems [15]. Even though we could not detect significant different results in respect to rotational stability of the two stems, there was a tendency towards more reversible and irreversible retrotorsion with the CBH bp stem compared to the CBH prosthesis. Comparing published results of micromotion measurements are challenging because of different test set-ups, loading conditions, and specimen used [16, 17]. However, results of in vitro primary stability investigations of the clinically proven CLS-type straight stem (Zimmer, Winterthur, Switzerland) with similar loading conditions showed comparable rotational motion to the CBH bp [3, 9]. Again, comparing the Zweymüller concept and a customized prosthesis with an extended shoulder showed higher resistance against torsional moments with the prosthesis and a more proximal fit-and-fill in an in vitro primary stability investigation confirming the results of the present study [18]. However, thinning of the proximal femoral bone stock especially the region of the greater trochanter might result in weakening of the abductor muscles as well as increasing the risk of perioperative trochanteric fractures [7, 19].

The study has several limitations: first, we lost two stems in each group before 100,000 loading cycles were applied. However, settling of the two stems happened far before the 40,000 loading cycles which could be applied in all cases. After the settling period, only small changes of micromotions, within the measurement accuracy, were detected. These findings are similar with earlier reports which described settling within 3000 and 8000 cycles with no significant changes afterwards [3]. Second, in vitro simulation simplify in vivo conditions and the single-leg stance might underestimate rotational forces [20]. Nevertheless, with the chosen position of the leg with adduction as well as flexion, a reasonable amount of torsional moment is applied, taking to some extent torsional moments into account that occur during stair climbing [21]. Furthermore, the percentage of stair climbing in daily activity is below 1 %, and the reproducibility is negatively influenced by testing more complex activities [20, 22].

## Conclusions

In conclusion, reducing the typical shoulder of the Zweymüller-type stem design does not negatively influence axial stability but might negatively influence rotational stability. Even though, comparable results still suggest a reasonable resistance against rotational forces.
